# Mitral annulus morphologic and functional analysis using real time tridimensional echocardiography in patients submitted to unsupported mitral valve repair

**DOI:** 10.5935/1678-9741.20140082

**Published:** 2015

**Authors:** Marco Antônio Vieira Guedes, Pablo Maria Alberto Pomerantzeff, Carlos Manuel de Almeida Brandão, Marcelo Luiz Campos Vieira, Flávio Tarasoutchi, Pablo da Cunha Spinola, Fábio Biscegli Jatene

**Affiliations:** 1 Instituto do Coração do Hospital das Clínicas da Faculdade de Medicina da USP (InCor HC-FMUSP), São Paulo, SP, Brazil.

**Keywords:** Mitral Valve, Mitral Valve Annuloplasty, Mitral Valve Insufficiency, Echocardiography, Echocardiography, Three-Dimensional

## Abstract

**Introduction:**

Mitral valve repair is the treatment of choice to correct mitral insufficiency, although the literature related to mitral valve annulus behavior after mitral repair without use of prosthetic rings is scarce.

**Objective:**

To analyze mitral annulus morphology and function using real time tridimensional echocardiography in individuals submitted to mitral valve repair with Double Teflon technique.

**Methods:**

Fourteen patients with mitral valve insufficiency secondary to mixomatous degeneration that were submitted to mitral valve repair with the Double Teflon technique were included. Thirteen patients were in FC III/IV. Patients were evaluated in preoperative period, immediate postoperative period, 6 months and 1 year after mitral repair. Statistical analysis was made by repeated measures ANOVA test and was considered statistically significant *P*<0.05.

**Results:**

There were no deaths, reoperation due to valve dysfunction, thromboembolism or endocarditis during the study. Posterior mitral annulus demonstrated a significant reduction in immediate postoperative period (*P*<0.001), remaining stable during the study, and presents a mean of reduction of 25.8% comparing with preoperative period. There was a significant reduction in anteroposterior and mediolateral diameters in the immediate postoperative period (*P*<0.001), although there was a significant increase in mediolateral diameter between immediate postoperative period and 1 year. There was no difference in mitral internal area variation over the cardiac cycle during the study.

**Conclusion:**

Segmentar annuloplasty reduced the posterior component of mitral annulus, which remained stable in a 1-year-period. The variation in mitral annulus area during cardiac cycle remained stable during the study.

## Abstract (pt)

**Introdução:**

A plastia valvar mitral é o tratamento de escolha para a insuficiência mitral, porém, a literatura é escassa em relação ao comportamento do anel mitral após a plástica mitral sem utilização de anéis protéticos.

**Objetivo:**

Realizar a análise morfofuncional do anel mitral de indivíduos submetidos à plastia valvar mitral pela Técnica de Duplo Teflon, sem utilização de anel protético, por meio da ecocardiografia tridimensional em tempo real.

**Métodos:**

Foram incluídos 14 pacientes com insuficiência mitral mixomatosa submetidos à plástica mitral pela técnica de Duplo Teflon. Treze pacientes encontravam-se em classe III/IV. Os pacientes foram avaliados nos períodos pré-operatório, pós-operatório imediato, 6 meses e 1 ano. Foi utilizado teste de análise de variância de medidas repetidas para o estudo estatístico, sendo considerado estatisticamente significante *P*<0,05.

**Resultados:**

Não houve óbito, reoperação por disfunção valvar, tromboembolismo ou endocardite durante o estudo. A planimetria posterior do anel mitral demostrou uma redução significativa (*P*<0,001) no pós-operatório imediato, que se manteve estável durante o estudo, apresentando redução média de 25,8% com 1 ano em relação ao pré-operatório. Houve uma redução significativa dos diâmetros ântero-posterior e médio-lateral no pós-operatório imediato (*P*<0,001), porém, houve um aumento significativo no diâmetro médio-lateral entre pós-operatório imediato e 1 ano. Não houve diferença na variação da área interna mitral ao longo do estudo.

**Conclusão:**

A anuloplastia segmentar reduziu significativamente o componente posterior do anel mitral, permanecendo estável no período de um ano. A variação da área valvar durante o ciclo cardíaco permaneceu estável durante o estudo.

**Table:** 

**Abbreviations, acronyms & symbols**	
AP	Anteroposterior
AP	Anteroposterior diameter
CI	Circularity index
FC	Functional class
ML	Mediolateral

## INTRODUCTION

Epidemiological data have demonstrated that mitral insufficiency secondary to prolapse of the valve, from moderate to severe extent, is the main valve disease in the United States^[1]^ and gives rise to the second most common form of surgically treated heart valve disease in Europe^[2]^. In Brazil, prolapse of the mitral valve was found to be the etiology of 25.9% of the patients undergoing mitral valvuloplasty, over the course of 12 years of experience at the Heart Institute of Hospital das Clínicas, University of São Paulo Medical School^[3]^.

Previous studies have demonstrated that conserving the mitral valve is better than replacing it^[4]^. In the technique of quadrangular resection of the posterior cuspid, with plication of the corresponding ring and edge-to-edge suturing of the cuspids, the use of prosthetic rings is still a matter for discussion^[5]^. In Brazil, Pomerantzeff et al.^[6]^ developed a technical modification in which threads with "pledgets" on a Teflon flap were used for segmental plication of the posterior ring corresponding to the segment removed from the cuspid, without using a prosthetic ring. This technique, named the "double Teflon technique", has presented excellent long-term results, with low morbidity and mortality rates^[7,8]^.

Three-dimensional is the diagnostic technique that has contributed most to knowledge of the anatomy and functioning of the mitral valve^[9]^. New discoveries regarding the saddle shape of the mitral valve ring^[10]^ and its dynamics during the cardiac cycle^[11]^ have provided great advances in the techniques for mitral valve conservation. The aim of the present study was to analyze the morphology and functioning of the mitral valve ring in individuals who underwent mitral valvuloplasty by means of the double Teflon technique, using real-time three-dimensional echocardiography.

## METHODS

**Fig. 1:**
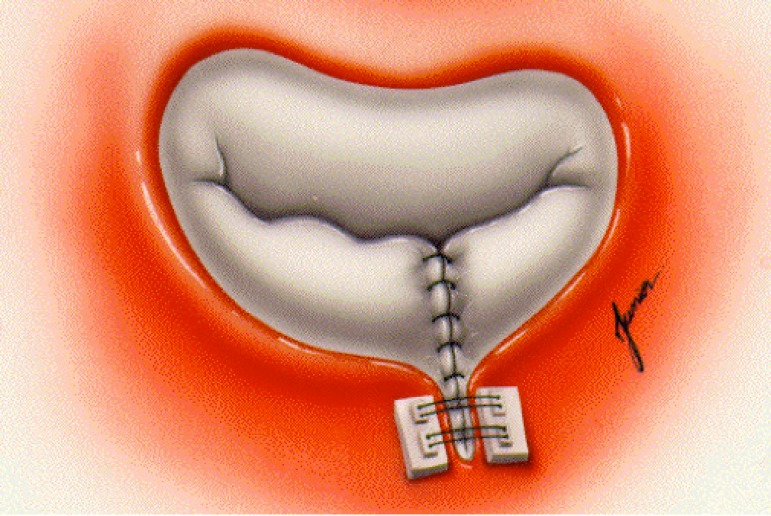
Intraoperative aspect of the mitral valve after completion of the mitral valve repair technique “Double Teflon”. We can observe the pledgets anchored in Teflon strips and the suture edge to edge of the cusps.

This study was conducted at the Heart Institute, Hospital das Clinicas, Faculty of Medicine, University of São Paulo for Heart Valve Surgery Unit and the Echocardiography Unit, with support from CEPEC (Echocardiography Research Center). After the study was approved by the Ethics Committee of Hospital das Clinicas, Faculty of Medicine, University of São Paulo and obtaining a written post-informed consent, between May/2006 and August/2008 we included 14 consecutive patients with mitral insufficiency secondary to mitral valve prolapse of degenerative etiology, due to elongation or tendon chordal rupture related to the mitral valve posterior cusp, who underwent mitral valve repair with the Double Teflon technique (Figure 1). Patients with associated valvular heart disease or submitted to previous heart surgery were excluded from the study. In this population, the age ranged between 39 and 75 years, with average of 61±11 years. Among all individuals, 10 were male and 4 were female. The average weight and height of patients was 75.6±10.9 kg and 1.69±0.1 m, respectively. Body surface area ranged between 1.64 and 2.10 m^2^, with a mean of 1.85±0.17 m^2^. In the investigation of personal history, 11 patients had hypertension; two patients had diabetes mellitus, two from chronic renal failure requiring dialysis, three had dyslipidemia, and two had coronary artery disease. The additive EuroSCORE ranged between 0 and 6, being that 11 cases presented additive EuroSCORE from 0 to 3 and the other 3 cases showed additive EuroSCORE between 3 and 6. Regarding the functional class (FC) in the preoperative period, one patient was in FC II, 12 in FC III and one in FC IV.

Among the 14 patients, eight had atrial fibrillation in the preoperative period. All patients were operated by the same surgical team. In one patient, plication of the free edge of the anterior mitral valve cusp was performed, as a technique associated with the repair. Regarding the location of mitral valve disease, 12 patients had involvement of the P2 segment, one patient had involvement of the P1 segment, and one patient had associated involvement of A2 and P2 segment.

Cord rupture were found in 10 patients, it was found string stretching in one patient; stretching and cord snapping found in two patients; calcification of the ring and cord rupture found in one patient. Two patients had coronary heart disease as an associated diagnosis. Of these patients, one patient had distal coronary lesion, not treatable surgically. One patient underwent revascularization of the left marginal branch.

Patients were evaluated in the preoperative period (up to 30 days before surgery), in the postoperative period (between 5 and 30 days after surgery), 6 months (between 6 and 7 months after surgery) and 1 year (between the 12^th^ and 15^th^month after surgery). In order to perform the test, the IE-33 (Philips Medical Systems, Andover, MA, USA) was used. Echocardiographic images were obtained using the matrix transducer positioned in the acoustic parasternal and apical windows.

The three-dimensional echocardiographic data were analyzed in a workstation, using specific software QLAB 5.0 and QLAB 6.0 (Philips Medical Systems, Andover, MA, USA). With the acquisition of three-dimensional data, the image was cut and reconstructed to visualize the cardiac structures within the pyramid or the whole heart block.

**Fig. 2:**
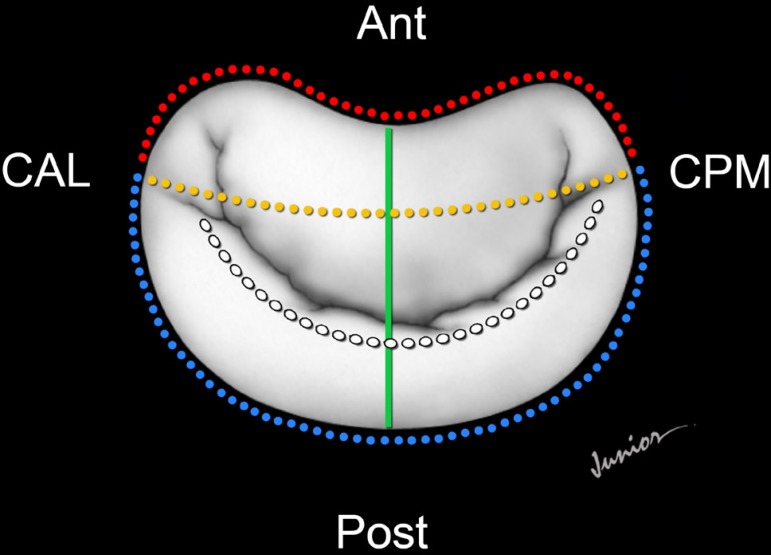
Illustration of the echocardiographic variables. Anterior perimeter=red line; posterior perimeter=blue line; anteroposterior diameter=green line; mediolateral diameter=yellow line. Ant=anterior; Post=posterior; CAL=anterolateral commissure, CPM=posteromedial commissure). The white line shows the line of valve coaptation.

Mitral annulus morphology was evaluated through anteroposterior diameter (AP), mediolateral (ML), annulus circunference, anterior and posterior mitral planimetry measurements (Figure 2).

Circularity index was obtained through the relationship of AP and ML diameter. Mitral annulus function was estimated between the difference of mitral valve area during maximum sistole and maximum diastole, in relationship to mitral valve area obtained at maximum sistole, described as valve area reduction during cardiac cycle.

In order to analyze the behavior of the group considering the conditions studied, we used the technique of Analysis of Variance for Repeated Measures. For the study of reproducibility of echocardiographic measurements we used the intraclass coefficient correlation. It was considered statistically significant *P*<0,05. The software SPSS version 15.0. (Inc, Chicago) was used for this analysis.

## RESULTS

During the study period, no death, endocarditis, reoperation for valve dysfunction or thromboembolism were observed. In terms of physical activity in the postoperative period, 12 patients were in functional class I and two in functional class II, one year after surgery. In the immediate postoperative period, 14 patients had mild mitral insufficiency. There was no significant change in the degree of mitral regurgitation after valvuloplasty during the study.

**Fig. 3:**
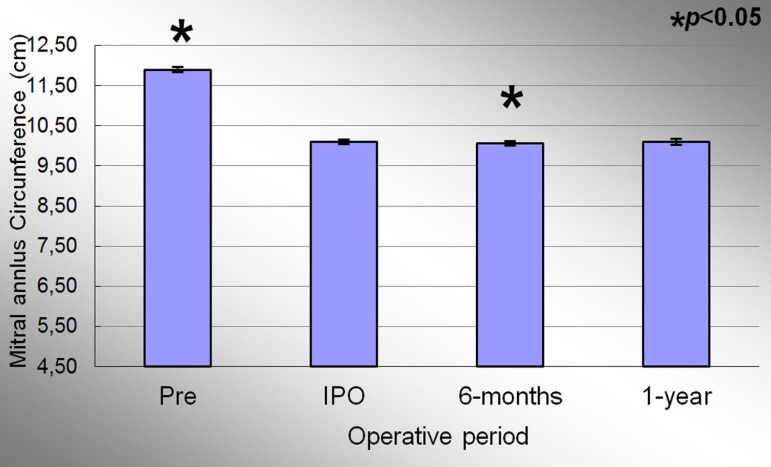
Evolution of the circumference of the mitral valve annulus during the study. Values =mean±standard deviation. *P<0.05 compared to the immediate postoperative period.

The mean circumference of the mitral annulus in the preoperative period, immediate post-operative period, 6 months and 1 year were 11.90±0.16cm; 10.10±0.13cm; 10.06±0.13cm and 10.10±0,13cm, respectively. Significant effect of condition evaluation was observed during the study period (*P*<0.001). At the end of the study, there was a decrease of 15.1% of the mitral annulus circumference when comparing the averages of the preoperative and 1 year period. Figure 3 represents the evolution of the mitral annulus circumference during the study. There was a decrease of 15.1% in the measurements when comparing the preoperative and IPO period (*P*<0.001). There was a 0.4% decrease in the circumference of the mitral annulus between the periods IPO and 6 months period (*P*=0<001). There was no difference between the IPO and 1 year period (*P*=1.0).

**Fig. 4:**
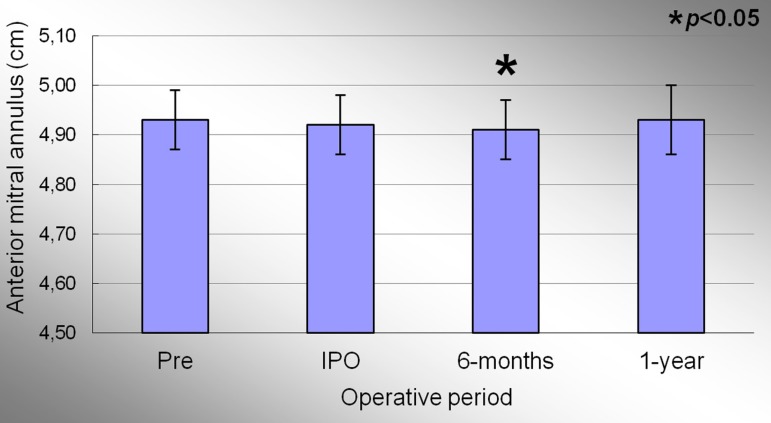
Evolution of the anterior perimeter of the mitral valve annulus during the study. Values =mean±standard deviation. *P<0.05 compared to the immediate postoperative period.

The anterior mitral annulus perimeter in the preoperative, immediate postoperative, 6 months and 1 year period were 4.93±0.06cm; 4.92±0.06cm; 4.91±0.06cm and 4.93±0.07cm, respectively. Significant effect of condition evaluation was observed during the study period (*P*<0.001). There was no difference in the average of the anterior perimeter in the preoperative and 1 year period. Figure 4 represents the evolution of the anterior perimeter of the mitral annulus during the study. When comparing the preoperative and immediate post-operative period there was no significant difference in anterior mitral annlus perimeter (*P*=0.17). There was a decrease in 0.2% between IPO and 6 months period (*P*<0.004). There was no statistical difference when comparing the IPO and 1 year period.

**Fig. 5:**
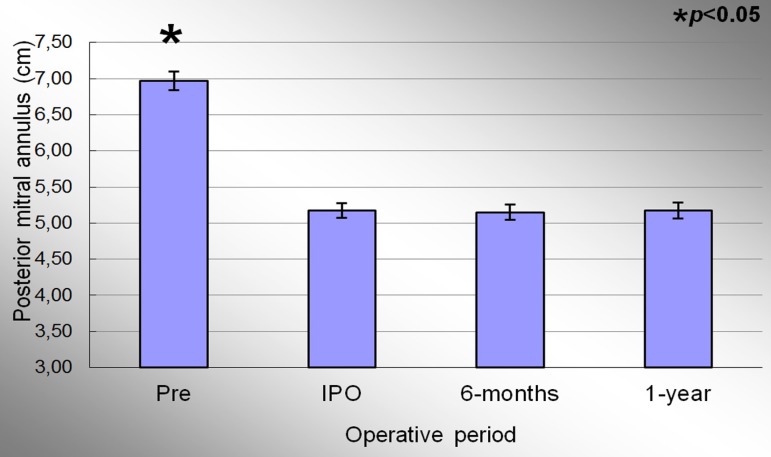
Evolution of the posterior perimeter of the mitral valve annulus during the study. Values =mean±standard deviation. *P<0.05 compared to the immediate postoperative period.

The posterior perimeter of the mitral annulus in the preoperative, immediate post-operative, 6 months and 1 year period were 6.97±0.13cm; 5.17±0.10cm, 5.15±0.11 and 5.17±0.11cm, respectively. Significant effect of condition evaluation was observed during the study period (*P*<0.001). At the end of the study, there was a decrease of 25.8% on the posterior perimeter of the mitral annulus betwwen preoperative and 1 year period. Figure 5 represents the evolution of the posterior mitral annulus perimeter throughout the study. When comparing the preoperative and IPO period, posterior mitral annulus decrease 25.8% (*P*<0.001), the same decrease value found when comparing the preoperative and 1 year period. There was a decrease in 0.2% between the IPO and 6 months period (*P*<0.003). There was no statistical difference when comparing the IPO and 1 year period (*P*=1.0).

**Fig. 6:**
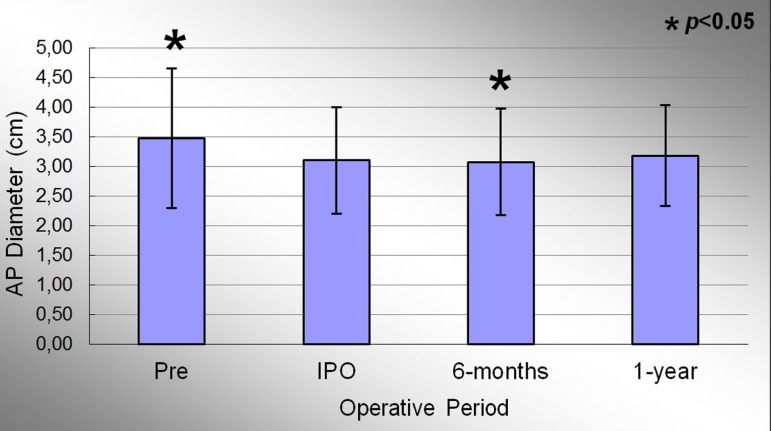
Evolution of antero-posterior diameter of the mitral valve annulus during the study. Values =mean±standard deviation. *P<0.05 compared to the immediate postoperative period.

The anteroposterior (AP) diameter of the mitral annulus in the preoperative, immediate post-operative, 6 months and 1 year period were 3.47±1.18cm; 3.10±0.90cm; 3.07±0.90cm and 3.18±0.85cm, respectively. Significant effect of condition evaluation was observed during the study period (*P*<0.001). At the end of the study, there was a decrease in AP diameter of 8.3% compared to the preoperative period. Figure 6 represents the evolution of AP diameter throughout the study. When comparing the preoperative and IPO period, there was evidenced a significant reduction in mean of this variable (*P*<0.001). When comparing the IPO and 6 months period, it was observed a slight further reduction of 1.0% in the mitral annulus AP diameter, statistically significant (*P*=0.012). The comparison between the IPO and 1 year period showed an increase in measures of this variable throughout the study, but without statistical significance (*P*=0.051).

**Fig. 7:**
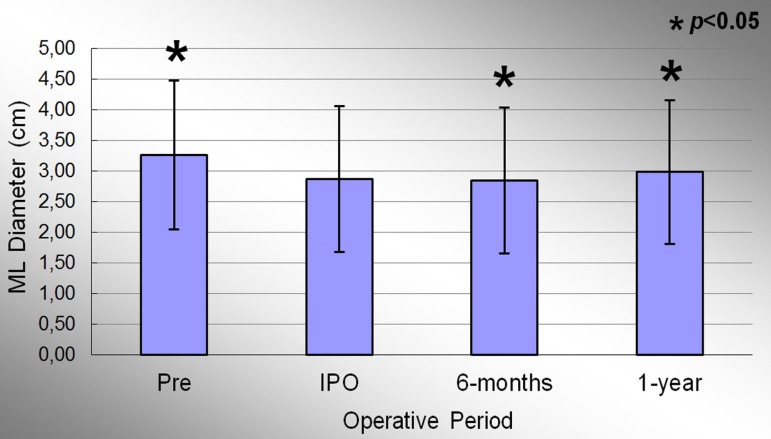
Evolution of the medial-lateral diameter of the mitral valve annulus during the study. Values =mean±standard deviation. *P<0.05 compared to the immediate postoperative period.

Mediolateral (ML) diameter of the mitral annulus in the preoperative, immediate post-operative, 6 months and 1 year period were 3.26±1.22cm, 2.87±1.19cm; 2.84±1.19cm and 2.98±0.17cm, respectively. Significant effect of condition evaluation was observed during the study period (*P*<0.001). At the end of the study, there was a decrease of 8.6% in the ML diameter of the mitral annulus when comparing the averages of the preoperative period. Figure 7 shows the evolution of the ML diameter the mitral valve annulus during the study. When comparing the preoperative and IPO period, There was evidenced a significant reduction in the average of this variable (*P*<0.001). When comparing the IPO and 6 months period, a slight further reduction of 1.0% was observed in the ML diameter, statistically significant (*P*=0.003). The comparison between the means of IPO and 1 year period showed an increase in measures of this variable by 3.8%, statistically significant (*P*=0.004).

Mitral annulus circularity index in preoperative, immediate post-operative, 6 months and 1 year period were 0.93±0.07, 0.91±0.13, 0.90±0.14 and 0.91±0.13, respectively. Significant effect of condition evaluation was not observed during the study period (*P*=0.59). The comparison between the means of POI and 1 year periods showed no difference between the means in these periods (*P*=0.29).

The reduction fraction of mitral internal area during cardiac cycle (variation of the internal area) in the preoperative period, immediate post-operative, 6 months and 1 year were 35.75±9.14%, 33.80±8.59%, and 33.90±8.91% 30.92±8.31%, respectively. Significant effect of condition evaluation was not observed during the study period (*P*=0.296). The comparison between the means of IPO and 1 year period showed a decrease of this variable by 8.5%, but without statistical significance (*P*=0.060).

The values of the intraclass correlation coefficient of the values obtained in the analysis of the posterior mitral annulus perimeter approached unity (1.0) in all analyzes (0.998 *P*<0.001).

Table 1 describes a subgroup analysis of the mitral valve annulus morphology variables taking into account the presence of atrial fibrillation during the study. There was no statistically significant difference in the behavior of the subgroups studied throughout the study, and the comparison of variable means in different times evaluated: circumference of the mitral annulus, anterior and posterior perimeter, AP and ML diameter, and circularity index.

**Table 1:** Subgroup analysis regarding the presence of atrial fibrillation.

Variable	Period	AF	No AF	*P*	
		(n=8)	(n=6)	P_A_	P_B_
CAM	Preoperative	11.90±0.18	11.90±0.16		
	IPO	10.03±0.08	10.19±0.13		
	6-months	10.00±0.07	10.15±0.15		
	1-year	10.03±0.08	10.19±0.15	0.471	0.051
Ant Annulus	Preoperative	4.91±0.07	4.96±0.05		
	IPO	4.90±0.06	4.95±0.06		
	6-months	4.89±0.06	4.93 ±0.06		
	1-year	4.91±0.06	4.95± 0.06	0.845	0.172
Post Annulus	Preoperative	6.99±0.12	6.94±0.13		
	IPO	5.13±0.06	5.23±0.13		
	6-months	5.11±0.05	5.21±0.14		
	1-year	5.13±0.05	5.23±0.13	0.553	0.086
AP Annulus	Preoperative	3.76±1.25	3.07±1.05		
	IPO	3.31±1.01	2.82±0.71		
	6-months	3.27±1.01	2.79±0.71		
	1-year	3.33±1.01	2.98±0.62	0.303	0.347
ML Annulus	Preoperative	3.53±1.30	2.90±1.10		
	IPO	3.22±1.26	2.41±1.01		
	6-months	3.17±1.26	2.39±1.02		
	1-year	3.29±1.28	2.56±0.93	0.166	0.264
CI	Preoperative	0.93±0.07	0.94±0.07		
	IPO	0.95±0.10	0.84±0.16		
	6-months	0.95±0.11	0.84±0.16		
	1-year	0.97±0.11	0.84 ± 0.13	0.143	0.155

Values=mean±standard deviation. P_A_=statistical significance comparing the groups with respect to behavior throughout the study, P_B_=statistical significance of the comparison of means between groups in different time periods. IPO=immediate postoperative; CAM=circumference of the mitral annulus; Ant Annulus=anterior mitral perimeter; Post Annulus=mitral posterior perimeter; AP Annulus=anteroposterior diameter; ML Annulus=medial-lateral diameter; CI=circularity index

## DISCUSSION

The mitral valve system is a complex structure. Clinical use of three-dimensional echocardiography has contributed significantly towards understanding its functioning and anatomy, especially with regard to the mitral valve ring^[11]^.

Fundaró et al.^[5]^ published a review of the most important studies that had analyzed the clinical results from annuloplasty techniques without a prosthetic ring. They classified the techniques as either mural or commissural annuloplasty. Mural annuloplasty techniques were subdivided into semicircular plication, when shortening of the entire posterior segment of the mitral ring is performed; and segmental plication, when plication is performed on the mitral ring corresponding to the segment of the posterior cuspid that is removed through quadrangular resection.

They found that the best immediate and late results occurred among patients with degenerative etiologies who had undergone segmental plication or semicircular reduction. In the immediate evaluation, the patients who had undergone these techniques presented low rates of residual mitral insufficiency, between 1 and 2%. In most of the studies reviewed, no early structural failure of the valve repair was observed. Medium-term evaluations showed good results. The actuarial five-year survival rate was approximately 90%. Moreover, the best survival results free from reoperation were found among patients who had undergone segmental plication or semicircular reduction, especially among those with degenerative mitral insufficiency. The actuarial survival curves free from thromboembolism and endocarditis presented excellent results. The authors concluded that the techniques of segmental and semicircular plication may be valid and safe options, especially for patients with prolapse of the posterior cuspid in association with slight dilatation of the mitral ring, thus reviving the doubts in relation to the need to use prosthetic rings^[5]^.

Brandão et al.^[8]^ obtained excellent clinical results from mitral valvuloplasty by means of the double Teflon technique, over a 10-year follow-up period. The actuarial survival rate was 94.1±3.6%, the survival rate free from thromboembolism was 97.3±1.5% and the survival rate free from reoperation was 99.2±0.8%.

A study on cadavers in which 712 valves resected from patients with mitral prolapse were examined showed that the mean ring circumference was 12.3 cm, whereas it was 9.8 cm in patients without annular dilatation^[12]^. In a study using three-dimensional echocardiography, Sonne et al.^[13]^ found great variation in the measurements of the mitral ring circumference among 123 normal individuals, with a mean of 10.5±1.4 cm and a range from 7.0 to 14.0 cm. In the present study, the mean circumference of the mitral ring before the operation was 11.9±0.16 cm, thus showing that the population studied had slight annular dilatation. This finding is probably related to the fact that most of the patients presented rupture of the tendinous cords as the genesis of their mitral insufficiency. After the surgical intervention, there was a significant reduction of 15.1% in the mitral circumference, attaining a mean value of 10.10±0.13, which then remained stable over the course of the follow-up.

The mitral ring was divided into two portions, taking the axis of the mediolateral (ML) diameter into consideration. Anatomically, the anterior portion of the mitral ring is composed of a fibrous portion that is located between the right and left trigones of the mitral ring, and two bilateral muscle portions that line between the ML axis and the corresponding trigone. In a study on cadavers, Hueb et al.^[14]^ found that the anterior intertrigonal distance of the mitral ring was greater in patients with dilated myocardiopathy. Suri et al.^[15]^ compared the anterior intertrigonal distance between patients with degenerative mitral insufficiency and normal individuals, using transesophageal three-dimensional echocardiography, and showed that there were no significant alterations of the anterior intertrigonal distance in these cases. These findings corroborate the idea that the mitral ring has different behavior according to the etiology of the mitral insufficiency. In the present study, planimetry on the anterior portion of the mitral ring showed that there was no significant variation over the study period.

The posterior segment of the mitral valve is formed by the muscle portion of the ring. In situations of degenerative etiology, there is annular dilatation corresponding to the posterior ring. Mihalatos et al.^[16]^ demonstrated that the degree of annular dilatation is directly related to the intensity of the mitral regurgitation, especially in patients with mitral prolapse and mitral functional insufficiency. Suri et al.^[15]^ found a mean posterior ring size that was greater than what we found in our study, and this is compatible with the predominance of rupturing of the tendinous cords in the present study. The segmental annuloplasty technique used in the present study significantly reduced the size of the posterior mitral ring, by 25.8%. This reduction remained stable over the course of the study period, and no annular redilatation was observed over this period. The double Teflon technique consists of plication of the anterior portion of the mitral ring alone, without interfering with its anterior portion. Therefore, the maintenance of the measurements of the anterior mitral ring and the reduction in the posterior ring that was found are compatible with the segmental annuloplasty technique that was applied in these cases.

Kwan et al.^[17,18]^ demonstrated that during the cardiac cycle, the variation in valve area is directly related to the increase in anteroposterior (AP) diameter of the mitral valve. This was not observed in relation to the ML diameter. The mitral valve becomes flatter at maximum systole, with increases in AP diameter and in the nonplanar angle, thus acquiring its greatest valve area. We analyzed the diameters at maximum systole and found values similar to those found by Kwan et al.^[18]^ in normal individuals. These findings confirm that the population studied here presented slight annular dilatation.

In pathological states, the annular dilatation seems not to be due to distension of the mitral ring fibers but, rather, due to an increase in the nonplanar angle, thereby modifying the shape of the mitral ring. This increase in the nonplanar angle gives the valve a more flattened appearance and seems to interfere more with the AP diameter than with the ML diameter.

In our study, we found that there were significant reductions in the measurements of the AP and ML diameters in the immediate postoperative period, compatible with the segmental annuloplasty. In comparing this time with one year after the operation, we found that these measurements had increased slightly: in absolute amounts, a mean of 0.8 mm for the AP diameter and a mean of 1.1 mm for the ML diameter. This change was probably related to the patients' hemodynamic status. Despite these slight increases from immediately after the operation to one year after the operation, we found that overall, there was a reduction of approximately 8% in the AP and ML diameters, from the preoperative diameters to the diameters at the end of the study period.

The circularity index (CI) is the ratio between the AP and ML diameters. The closer that this is to 1.0, the more circular the shape of the mitral valve is. Mahmood et al.^[19]^ compared the nonplanar angle and the CI in patients who had undergone mitral valvuloplasty in which prosthetic rings were used. Out of the 75 patients studied, 40 of them presented degenerative etiologies. In the same study, eight patients were used as controls and their normal mitral valves presented a CI of 0.90. In our study, we found a CI of 0.93 before the operation, which was similar to what Mahmood et al.^[19]^ found in normal valves. Moreover, we did not find any significant changes to this index over the course of the study, thus showing that the proportions between the AP and ML diameters were maintained at the different measurement times of the study. Mahmood et al.^[19]^ also demonstrated that prosthetic ring implants may alter both the nonplanar angle and the CI, thereby changing the saddle shape of the mitral ring, and that prosthetic rings have different behavior in remodeling the mitral ring.

The measurements of the internal area of the circumference obtained through planimetry can be made both at the end of the systole phase and at the end of the diastole phase, thus enabling comparison of the variation of the internal area during the cardiac cycle. This methodology for measuring the valve area presents the limitation of being a two-dimensional measurement of a three-dimensional structure. It consists of a projection of the three-dimensional mitral ring structure into a transverse plane. Therefore, simple changes to the nonplanar angles would have an impact on the accuracy of the method.

In relation to the mitral ring area and the magnitude of the variation of this area during the cardiac cycle, the results described in the literature have been diverse, particularly in clinical studies. We believe that these findings relate mainly to lack of standardization of the times at which measurements were made in different studies, thus causing difficulty in making comparisons between them. Moreover, anatomical evaluations of the mitral ring through echocardiography make use of the insertion site of the mitral cuspid, which does not necessarily reflect the exact intramuscular location of the mitral ring. Nonetheless, the dynamic nature of the mitral ring during the cardiac cycle has been well established, both in experimental studies and in clinical studies. In these studies, the maximum reduction of the mitral ring size in normal individuals during the cardiac cycle has been shown to be between 22-35%^[17,18]^.

Analyses on mitral ring dynamics in relation to valvuloplasty using a prosthetic ring has given rise to divergences in the literature. Okada et al.^[20]^ observed a variation in valve area during the cardiac cycle of 26±4%, among patients who underwent implantation of a flexible Duran ring (Duran; Medronic Heart Valve Division, Minneapolis, MN, USA). However, there was no variation in valve area when a rigid Carpentier ring was implanted (Carpentier-Edwards [C-E] Physio; Edwards Lifesciences Corp, Irvine, CA, USA), thus demonstrating behavior that was more physiological than that of the flexible ring. Gillinov et al.^[21]^ evaluated the Cosgrove partial flexible ring (Cosgrove-Edwards annuloplasty band; Edwards Lifesciences Corp, Irvine, CA, USA) and showed that this prosthesis maintained the saddle shape of the mitral ring, and also presented variation in the valve area of 28±11%, five years after implantation.

Implantation of prosthetic rings interferes with the saddle shape of the ring. Mahmood et al.^[19]^ showed that implantation of a complete ring interfered with the nonplanar angle, both in patients with ischemic and in patients with degenerative mitral insufficiency. Furthermore, different behavior was observed according to the type of partial ring used in the study, thus suggesting that morphological analysis on the mitral valve after implantation might influence the choice of device.

Komoda et al.^[22]^ showed that there was a reduction in the contraction of the base of the left ventricle after fixation of the mitral ring by means of prosthetic rings. Moreover, in a study using magnetic resonance, it was demonstrated that mitral plastic techniques that did not involve using prosthetic rings did not interfere with the contraction of either the mitral valve or the base of the left ventricle, as observed six months after the surgical intervention. In the present study, we observed that after the surgical intervention, there was no significant reduction in mitral ring performance, thus showing that the segmental annuloplasty did not have any significant impact on mitral ring function and that the ring continued to function in a stable manner over the course of the study, such that the mitral ring maintained dynamics that were more physiological.

Barlow disease is generally found in young patients and is characterized by myxomatous degeneration affecting the entire valve, thereby resulting in excess of tissue in the cuspids and leading to redundant tissue, with prolapse in different segments of the valve. Surgical intervention is generally required in the fifth or sixth decade of life. Because of the long course of this disease, it is usually associated with significant dilatation of the mitral ring. On the other hand, fibroelastic deficiency is found in patients over the age of 60 years who present a rapid course of mitral valve disease. Fibroelastic deficiency is a disease that essentially affects the tendinous cords and not the cuspids of the mitral valve, and it predisposes towards rupture of the tendinous cords, generally in a single segment of the valve. This condition can be diagnosed by means of preoperative echocardiography findings that suggest that the valve is of normal size, with thin cuspids and little excess tissue, seen in association with rupturing of the tendinous cords, generally in the P2 segment of the mitral valve. Although Barlow disease and fibroelastic deficiency present different characteristics, these conditions cannot be distinguished in approximately 20% of the patients^[23]^. In our series, we found echocardiographic characteristics that were compatible with fibroelastic deficiency in the majority of our patients. Our patients presented prolapse of a single segment, affecting the P2 segment in 80% of the cases, in association with slight dilatation of the mitral valve.

We believe that the decision to use prosthetic rings should be correlated with ventricular function and the size of the mitral ring. It is possible that patients with normal ventricular function and slight annular dilatation (the characteristics observed in cases of fibroelastic deficiency) would benefit from correction of their mitral insufficiency through techniques that do not involve using prosthetic rings. On the other hand, patients with ventricular dysfunction and significant dilatation of the mitral ring might benefit from techniques involving use of prosthetic rings for remodeling and stabilizing the mitral ring, because their disease affects not only the mitral valve but also the left ventricle.

Atrial fibrillation is an independent predictor of cardiovascular events^[24]^. In this series, approximately half of the patients presented this arrhythmia over the course of the study. We conducted a subgroup analysis to evaluate whether the presence of atrial fibrillation might have had an impact on the results found from this study. We did not observe any significant alterations to the behavior of the variables evaluated over the course of the study, thus demonstration that the mitral valvuloplasty modified the ring measurements in a stable manner and also enabled reverse atrial and ventricular remodeling, independent of atrial fibrillation^[25]^. It is possible that the patients who persisted with atrial fibrillation over the course of time presented differences not in relation to behavior but, rather, in relation to the magnitude of the remodeling. However, the results obtained should be viewed with caution because of the small number of patients allocated to each group in this analysis.

Although this study had a sample of 14 patients, there is little data in the literature regarding remodeling of the mitral valve ring during the postoperative period. Moreover, the populations studied have been etiologically different from each other and they generally underwent mitral valvuloplasty by means of techniques that involved usie of prosthetic rings. The present study described aspects of the morphology and functioning of the mitral ring over the course of a one-year postoperative period, in a population that was homogenous with regard to the etiology of the mitral insufficiency, which underwent mitral valvuloplasty without involving the use of prosthetic rings.

## CONCLUSION

We conclude that the patients who underwent mitral valvuloplasty by means of the double Teflon technique presented reductions in the posterior segment of the mitral ring, and that this remained stable over the one-year period. Moreover, the variation in internal valve area during the cardiac cycle remained stable over the course of the study.

**Table:** 

**Authors’ roles & responsibilities**	
MAVG	Manuscript writing or critical review of its content
PMAP	Analysis and/or interpretation of data; final approval of the manuscript; study design; implementation of projects and/ or experiments; manuscript writing or critical review of its content
CMAB	Analysis and/or interpretation of data; final approval of the manuscript; study design; implementation of projects and/ or experiments; manuscript writing or critical review of its content
MLCV	Analysis and/or interpretation of data; study design; imple-mentation of projects and/or experiments; manuscript writing or critical review of its content
FT	Final approval of the manuscript; implementation of projects and/or experiments
PCS	Performed operations and/or experiments
FBJ	Analysis and/or interpretation of data; final approval of the manuscript; study design; manuscript writing or critical review of its content
